# PRELIMINARY FINDINGS ON FAMILY PERPETRATORS AS DESIGNATED SURROGATE DECISION MAKERS

**DOI:** 10.1093/geroni/igz038.1407

**Published:** 2019-11-08

**Authors:** Cory Bolkan, Pamela Teaster, Holly Ramsey-Klawsnik, Kenneth Gerow

**Affiliations:** 1 Washington State University, Vancouver, Washington, United States; 2 Virginia Tech, Blacksburg, Vermont, United States; 3 National Adult Protective Services Association, Washington, District of Columbia, United States; 4 University of Wyoming, Laramie, Wyoming, United States

## Abstract

Vulnerable older adults needing surrogate decision makers typically rely upon others for care and are unable to advocate for themselves. The issue of EFFE perpetrated by family members designated as surrogates has become highly visible nationally, yet no reliable, empirical documentation exists on the nature or extent of exploitation by surrogate perpetrators. In collaboration with the National Adult Protective Services Association (NAPSA), we prospectively gathered APS data from six geographically diverse counties on 450 substantiated cases of abuse by POAs, representative payees, and guardians of vulnerable adults 65+ living in community settings. This presentation will highlight how family member surrogates perpetuated abuse and the outcomes on elder victims. These findings elucidate person and process-level factors (e.g., characteristics of victims, perpetrators, and their relationships) within the context of the APS system and can inform practice and policy recommendations for better prevention, detection, investigation, and intervention in these challenging cases.

